# Economic evaluation of anlotinib plus penpulimab vs. sorafenib as first-line therapy for unresectable hepatocellular carcinoma in China

**DOI:** 10.3389/fpubh.2025.1634266

**Published:** 2025-12-01

**Authors:** Rui Fang, Jiajun Liang, Tieqiao Wang, Feifeng Sheng, Jun Xu

**Affiliations:** 1 Department of Pharmacy, Guangdong Women and Children Hospital, Guangzhou, Guangdong, China; 2 Information Center, Guangdong Women and Children Hospital, Guangzhou, Guangdong, China; 3 College of Pharmacy, Jinan University, Guangzhou, Guangdong, China

**Keywords:** cost-effectiveness analysis, partitioned survival model, anlotinib, penpulimab, sorafenib, unresectable hepatocellular carcinoma

## Abstract

**Introduction:**

While the APOLLO trial confirmed the clinical efficacy of first-line anlotinib plus penpulimab in unresectable hepatocellular carcinoma (HCC), its economic impact on China’s healthcare system remains underexplored. This study was conducted to evaluate the cost-effectiveness of this novel combination vs. sorafenib from the perspective of the Chinese healthcare system.

**Methods:**

A partitioned survival model with three health states was developed to simulate economic outcomes for patients with advanced HCC. Survival data were derived from the APOLLO trial using parametric fitting. Direct medical costs and utility values were obtained from local public databases and the published literature. The primary outcomes included total costs, quality-adjusted life years (QALYs), and incremental cost-effectiveness ratios (ICERs) evaluated against the willingness-to-pay (WTP) threshold of $40334.05/QALY. Model robustness was assessed through deterministic and probabilistic sensitivity analyses (PSA).

**Results:**

The base-case analysis revealed that anlotinib plus penpulimab incurred a total cost of $25681.69 and yielded 1.42 QALYs, compared with sorafenib’s total cost of $18082.48 and 1.19 QALYs. This resulted in an incremental cost of $7599.21 and an incremental effectiveness of 0.22 QALYs, resulting in an ICER of $34050.28/QALY, which is below the predefined WTP threshold. Sensitivity analyses identified anlotinib treatment duration (cycles) and progression-free survival (PFS) utility values as key drivers of model variability. The PSA indicated an 85.9% probability of cost-effectiveness at the WTP threshold.

**Conclusion:**

Anlotinib plus penpulimab represents a potentially cost-effective first-line treatment for advanced HCC from a Chinese healthcare system perspective. These findings support incorporating this regimen into guidelines for selecting cost-effective immunotherapeutic strategies and provide evidence to inform decision-making about resource allocation for advanced HCC management.

## 1 Introduction

Hepatocellular carcinoma (HCC), a major global health burden, is the third most prevalent cause of cancer mortality worldwide (1). In China, it represents a significant epidemiological burden, ranking as the fourth most common cancer and the second leading cause of cancer-related death. Despite advances in early detection, 60–70% of patients are diagnosed with advanced disease, making curative interventions (e.g., surgical resection, ablation, or transplantation) infeasible (2, 3). For over a decade, tyrosine kinase inhibitors (TKIs), such as sorafenib and lenvatinib, have been the standard first-line treatment, modestly extending median overall survival (OS) to 10–15 months (4, 5). However, therapeutic advances remain limited, and the 5-year survival rate for advanced HCC continues to be poor.

The emergence of immune checkpoint inhibitors (ICIs), particularly PD-1/PD-L1 inhibitors such as nivolumab, pembrolizumab, sintilimab, and penpulimab, has transformed the therapeutic paradigm by significantly improving OS in patients with unresectable HCC (6). Although single-agent ICIs have not demonstrated OS improvements over sorafenib (7), combination strategies have emerged as a focal point of research. Notable regimens include atezolizumab plus bevacizumab (IMbrave150), cabozantinib plus atezolizumab, sintilimab plus IBI305 (ORIENT-32), camrelizumab plus rivoceranib (CARES-310), pembrolizumab plus lenvatinib (LEAP-002), and durvalumab plus tremelimumab (HIMALAYA) (8–14).

The APOLLO trial demonstrated that first-line penpulimab, a novel humanized immunoglobulin (Ig) G1 antibody with a high affinity for PD-1 (15), plus anlotinib, a small-molecule, multi-target TKI (16), significantly improved median progression-free survival (PFS: 6.9 vs. 2.8 months; HR 0.52, 95% CI 0.41–0.66) and median overall survival (OS: 16.5 vs. 13.2 months; HR 0.69, 95% CI 0.55–0.87) compared to sorafenib, with manageable grade ≥3 adverse events (56.5% vs. 55.1%) (17). These results indicate that this regimen is a promising first-line option for advanced HCC.

Despite its clinical efficacy, the economic implications of penpulimab plus anlotinib have not been thoroughly evaluated in the context of constrained healthcare resources. To date, no cost-effectiveness analysis has assessed this regimen for advanced HCC in China. This study aims to evaluate the cost-effectiveness of anlotinib plus penpulimab vs. sorafenib from the perspective of the Chinese healthcare system, providing essential evidence to support value-based treatment guidelines and inform health policy.

## 2 Methods

### 2.1 Model overview

The target population consisted of patients with unresectable HCC who had received no previous systemic therapy, consistent with the patient characteristics in the APOLLO trial.

**Figure 1:**
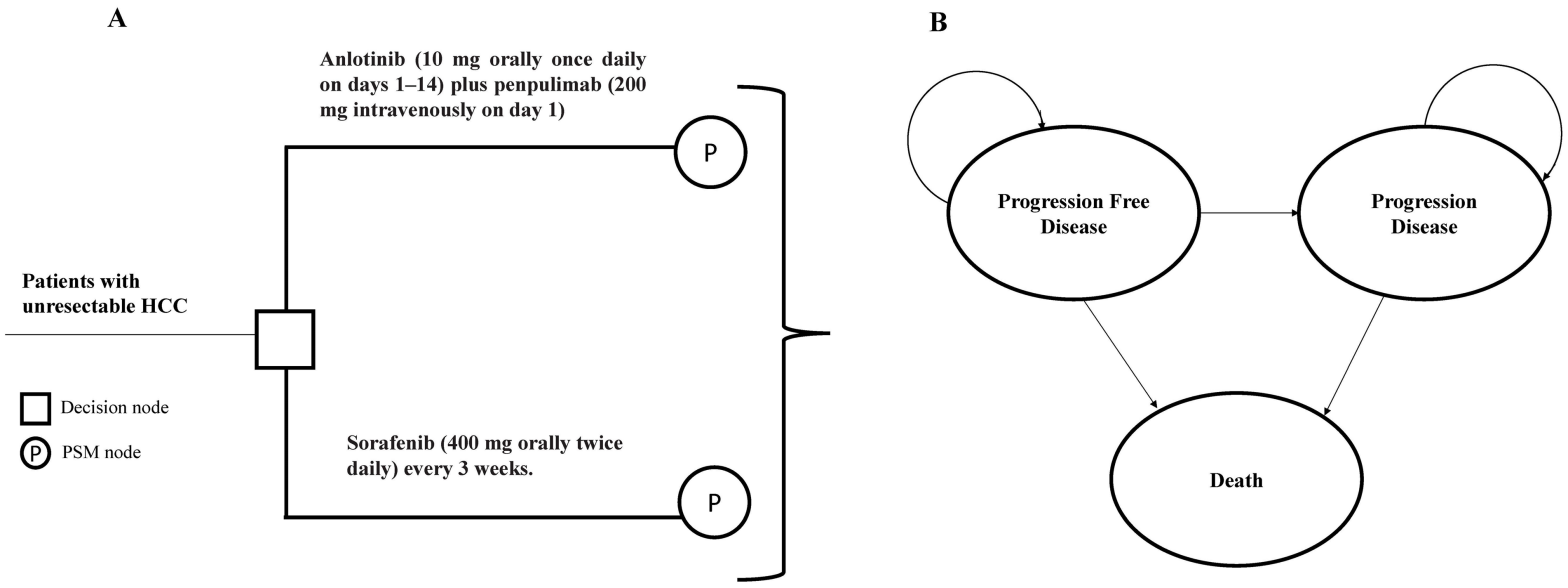
Model structure of the partitioned survival model with three health states. **(A)** Decision tree used to compare the standard first-line treatment for patients with HCC. **(B)** The PSM model simulated three health states: progression-free survival, progressive disease, and death. HCC, hepatocellular carcinoma; PSM, partitioned survival model.

A partitioned survival model (PSM) was developed using TreeAge Pro 2022 (TreeAge, Williamstown, MA, United States) to assess the cost-effectiveness of anlotinib plus penpulimab compared with sorafenib in advanced HCC (Figure 1). The PSM approach was selected because it allows direct extrapolation of the Kaplan–Meier curves for OS and PFS from the APOLLO trial. This finding offers a major advantage by accurately reflecting observed patient outcomes without requiring assumptions regarding transition probabilities between health states—a necessity in Markov models. Given that the treatment benefit of anlotinib plus penpulimab is characterized by concurrent improvements in both PFS and OS, the PSM provides a more direct and less assumption-dependent representation of this combined treatment effect.

The model consisted of three mutually exclusive health states: PFS, progressive disease (PD), and death. The model cycle was set to 21 days, consistent with the therapeutic regimens used in pivotal trials, with a 10-year simulation time horizon, reflecting both the poor 5-year OS rate of 20% for advanced HCC in China (18) and the follow-up duration of the APOLLO trial.

### 2.2 Clinical data inputs

Individual patient survival data were digitized from the Kaplan–Meier curves in the APOLLO trial using GetData Graph Digitizer (version 2.26) and reconstructed in R (version 4.5.0, Vienna, Austria) (Supplementary Table S1). Six parametric models (exponential, Weibull, log-normal, log-logistic, Gompertz, and generalized gamma) were fitted and used to extrapolate survival curves beyond the trial follow-up period. Model selection was guided by a combination of visual inspection and statistical criteria, including the Akaike Information Criterion (AIC) and Bayesian Information Criterion (BIC), with the log-normal distribution demonstrating optimal fit for simulating the survival curves (Supplementary Table S2; Supplementary Figures S1, S2). The optimal log-normal model parameters are summarized in Table 1.

**Table 1:** Log-normal survival model parameters for PFS and OS.

Variables	Mean	SE	95% CI	
			Low	Up
Survival model for anlotinib plus penpulimab				
Log-normal model for PFS	meanlog = 1.884	0.069	1.749	2.019
	sdlog = 1.078	0.054	0.977	1.189
Log-normal model for OS	meanlog = 2.836	0.062	2.714	2.958
	sdlog = 1.042	0.053	0.943	1.153
Survival model for sorafenib				
Log-normal model for PFS of	meanlog = 1.348	0.080	1.192	1.503
	sdlog = 0.899	0.062	0.785	1.029
Log-normal model for OS	meanlog = 2.509	0.095	2.322	2.696
	sdlog = 1.193	0.082	1.043	1.366

PFS, progression-free survival; OS, overall survival.

### 2.3 Cost and utility inputs

Direct medical costs comprised medications, management of serious adverse events (AEs), laboratory tests and radiological examinations, routine follow-up, subsequent systemic therapies, drug administration, end-of-life care, and best supportive care (Table 2). Treatment duration and dosing schedules are provided in Supplementary Table S3. Specifically, drug prices were obtained from the Chinese bid-winning price data in the Yaozhi database, a big-data service provider for the Chinese healthcare industry (19). Penpulimab is priced at approximately $1006.31 per cycle; however, a patient assistance program (PAP) offers a “4 + 2” scheme (four cycles purchased and two cycles provided free) for the initial phase, followed by a “treatment until progression” model after the next two purchased cycles (two cycles purchased and then provided free thereafter). Based on an average of 10 treatment cycles, the effective cost per cycle is reduced to approximately $603.79. Costs of administration, laboratory tests, and radiological examinations, routine follow-up, and best supportive care per cycle were obtained from the Guangdong Provincial Medical Insurance Bureau, the provincial government agency responsible for planning, policy-making, and oversight of the medical insurance system, as well as for organizing public tendering and procurement of drugs and medical consumables (20).

**Table 2:** Key clinical and health preference data.

Variables	Base value	Range		Distribution	Source
		Minimum	Maximum		
Costs, per cycle ($)					
Penpulimab (200 mg)	603.79	483.03	724.55	Gamma	(19)
Anlotinib (10 mg)	564.20	451.36	677.04	Gamma	(19)
Sorafenib (200 mg)	265.44	212.35	318.53	Gamma	(19)
Administration cost	127.40	101.92	152.88	Gamma	(20)
Cost of laboratory tests and radiological examinations	672.02	537.62	806.42	Gamma	(20)
Subsequent systemic therapy in sorafenib group	330.09	264.07	396.11	Gamma	(57)
Subsequent systemic therapy in anlotinib plus penpulimab group	272.68	218.14	327.22	Gamma	(57)
Routine follow-up cost	73.57	58.86	88.28	Gamma	(20)
Best supportive care	274.00	219.20	328.80	Gamma	(20)
End-of-life care per month	1460.30	1168.24	1752.36	Gamma	(21)
Costs of serious adverse events per time ($)					
Hypertension	0.17	0.14	0.21	Gamma	(22)
Platelet count decreased	1640.63	1312.50	1968.75	Gamma	(22)
Aspartate aminotransferase concentrations increased	33.31	26.65	39.97	Gamma	(22)
Blood bilirubin concentrations increased	124.90	99.92	149.88	Gamma	(58)
White blood cell counts decreased	126.39	101.11	151.67	Gamma	(51)
Neutrophil count decreased	126.39	101.11	151.67	Gamma	(51)
Palmar-plantar erythrodysesthesia syndrome	145.65	116.52	174.78	Gamma	(23)
Duration of treatment					
Duration of treatment penpulimab	10.00	8.00	12.00	Gamma	(17)
Duration of treatment anlotinib	10.00	8.00	12.00	Gamma	(17)
Duration of treatment sorafenib	4.00	3.20	4.80	Gamma	(17)
Risk of Grade ≥ 3 TRAEs in anlotinib plus penpulimab group (%)					
Hypertension	17.36	13.89	20.83	Beta	(17)
Platelet count decreased	9.03	7.22	10.83	Beta	(17)
Aspartate aminotransferase concentrations increased	4.17	3.33	5.00	Beta	(17)
Blood bilirubin concentrations increased	5.32	4.26	6.39	Beta	(17)
White blood cell counts decreased	5.56	4.44	6.67	Beta	(17)
Neutrophil count decreased	5.79	4.63	6.94	Beta	(17)
Palmar-plantar erythrodysesthesia syndrome	1.85	1.48	2.22	Beta	(17)
Risk of grade ≥ 3 TRAEs in sorafenib group (%)					
Hypertension	10.43	8.34	12.51	Beta	(17)
Platelet count decreased	6.16	4.93	7.39	Beta	(17)
Aspartate aminotransferase concentrations increased	6.16	4.93	7.39	Beta	(17)
Blood bilirubin concentrations increased	2.37	1.90	2.84	Beta	(17)
White blood cell counts decreased	3.32	2.65	3.98	Beta	(17)
Neutrophil count decreased	3.32	2.65	3.98	Beta	(17)
Palmar-plantar erythrodysesthesia syndrome	8.06	6.45	9.67	Beta	(17)
Utility value and discount rate					
PFS	0.76	0.61	0.91	Beta	(22)
PD	0.68	0.54	0.82	Beta	(22)
Discount rate	0.03	0	0.08	Fixed	(59)

PFS, progression-free survival; PD, progressive disease.

For adverse events, grade ≥3 AEs with ≥5% incidence in the APOLLO trial were included, such as increased AST levels, decreased white blood cell count, decreased neutrophil count, bilirubin elevation, decreased platelet count, palmar-plantar erythrodysesthesia syndrome, and hypertension. Treatment-related grade ≥3 AEs from the APOLLO trial are listed in Supplementary Table S4. The risk of TRAEs in both treatment groups is shown in Table 2, with AE management costs and subsequent systemic therapy costs referenced from the literature (21–23). The subsequent treatments for patients in the PD state were aligned with those used in the APOLLO trial. The best supportive care was applied continuously from the point at which a patient exited active anti-cancer therapy (either after progression or upon discontinuation for toxicity) until death. End-of-life costs were calculated for patients in their final month of life. All costs were adjusted and presented in 2024 USD (1 USD = 7.1217 RMB) and adjusted for inflation using the annual Consumer Price Index for healthcare published by the National Bureau of Statistics of China (CPI = 100.6 for 2022, 101.1 for 2023, and 101.3 for 2024) (24, 25).

Health-related quality of life was measured using utility values (0 = death, 1 = perfect health). Due to limited data in the APOLLO, PFS, and PD utilities (0.76 and 0.68, respectively), they were adapted from the literature (22). According to the Chinese Pharmacoeconomic Guidelines (2020) and WHO standards (26, 27), all inputs were discounted at 3%, with a willingness-to-pay (WTP) threshold of three times China’s per capita gross domestic product (GDP) in 2024 ($40334.05) (28).

### 2.4 Sensitivity analysis

Model robustness was evaluated through a one-way sensitivity analysis and probabilistic sensitivity analysis (PSA). A one-way sensitivity analysis was performed by varying all input parameters based on the estimated 95% confidence intervals or a ± 20% change from the base-case value (Table 2). PSA involved 1,000 Monte Carlo simulations by sampling key model parameters from predefined distributions to reflect their inherent variability. A gamma distribution was selected for the cost parameters and a beta distribution for the probability, proportion, and preference value parameters. Cost-effectiveness acceptability curves and a probabilistic scatter plot were generated to illustrate the probability of the intervention being cost-effective across WTP thresholds.

### 2.5 Scenario analysis

In scenario 1, we conducted a sensitivity analysis by varying the model’s time horizon to 1, 2, 5, and 10 years to evaluate the impact on the results. In scenario 2, we evaluated a situation without the Patient Assistance Program (PAP), incorporating the full drug cost of penpulimab at approximately $1006.31 per cycle.

## 3 Results

### 3.1 Base-case analysis

The base-case analysis showed that anlotinib plus penpulimab is cost-effective compared with sorafenib for advanced HCC (Table 3). The total direct medical cost was $25681.69 for the anlotinib plus penpulimab group vs. $18082.48 for the sorafenib group. The difference in total direct cost was primarily driven by the cost of anlotinib plus penpulimab, which accounted for 30% of the total cost (Supplementary Table S5). After adjusting for health-state utilities, anlotinib plus penpulimab yielded 1.42 QALYs vs. 1.19 QALYs for sorafenib, representing an incremental effectiveness of 0.22 QALYs. The ICER was $34050.28 per QALY, below the WTP threshold of $40334.05/QALY, establishing the regimen as potentially cost-effective from the perspective of the Chinese healthcare system.

**Table 3:** Summary of cost and outcome results of the cost-effectiveness analysis.

Treatment	Total cost ($)	QALY	Incremental cost ($)	Incremental QALY	ICER ($/QALY)
Anlotinib plus penpulimab	25681.69	1.42	7599.21	0.22	34050.28
Sorafenib	18082.48	1.19			

QALY, quality-adjusted life year; ICER, incremental cost-effectiveness ratio.

### 3.2 Sensitivity analyses

**Figure 2:**
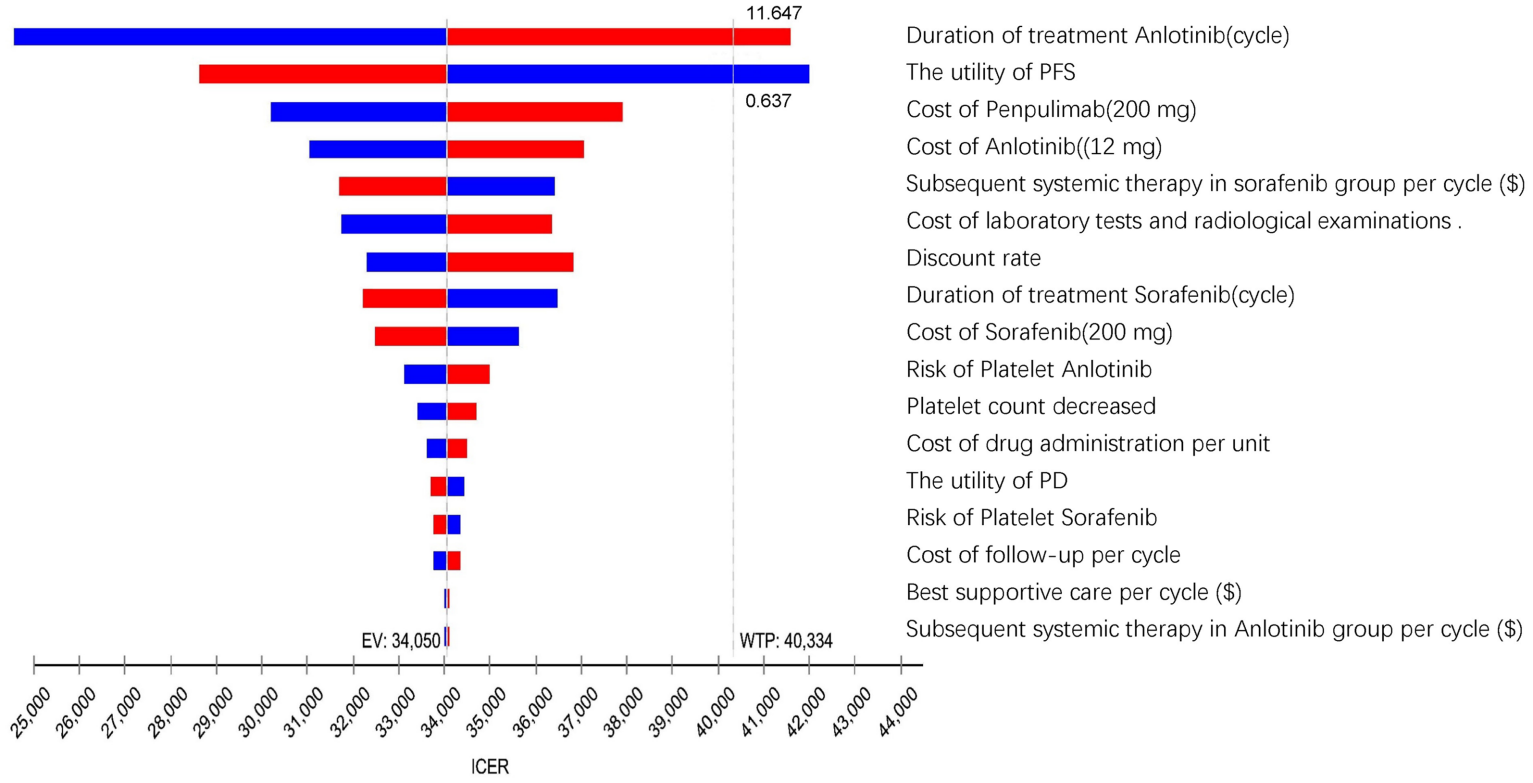
The tornado diagram summarizes the result of the one-way sensitivity analysis. QALY, quality-adjusted life year; GDP, gross domestic product; ICER, incremental cost-effectiveness ratios; WTP, willingness to pay; EV, expected value; PFS, progression-free survival; PD, progressive disease.

The one-way sensitivity analysis results, visualized in a tornado diagram (Figure 2), identified the treatment duration of anlotinib (cycles) and the utility of PFS as the most influential parameters. A ± 20% change from base-case values generated ICER ranges of $24549.20–$41595.25 per QALY (for treatment duration) and $28632.00–$41997.89 per QALY (for PFS utility). In the tornado diagram, the ICER exceeded the WTP threshold only when anlotinib cycles exceeded 11.64 or PFS utility fell below 0.637. Other considerably influential factors included the cost of penpulimab and anlotinib, the cost of subsequent therapy in the sorafenib group, the cost of laboratory tests and radiological examinations, the discount rate, the duration of sorafenib treatment, the cost of sorafenib, the risk of decreased platelet count with anlotinib, and the cost of drug administration. However, even with variations in these parameters within the predetermined range, the ICER consistently remained below the WTP threshold.

**Figure 3:**
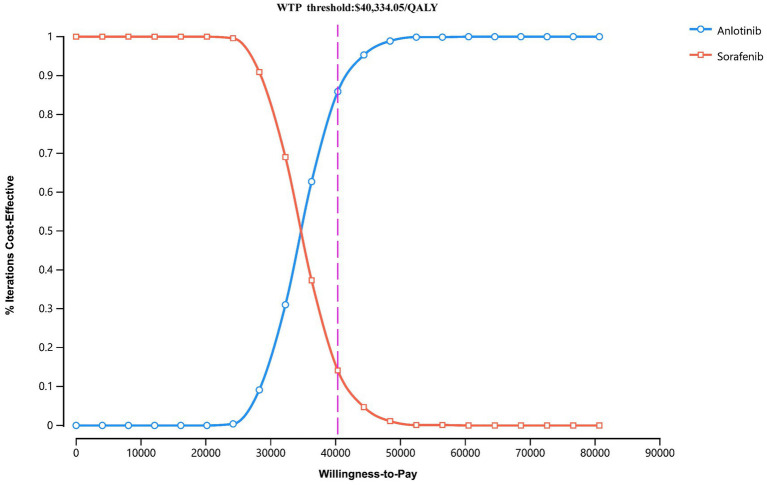
Cost-effectiveness acceptability curves for anlotinib plus penpulimab vs. sorafenib. The purple dashed lines indicate the willingness-to-pay threshold. WTP, willingness-to-pay.

**Figure 4:**
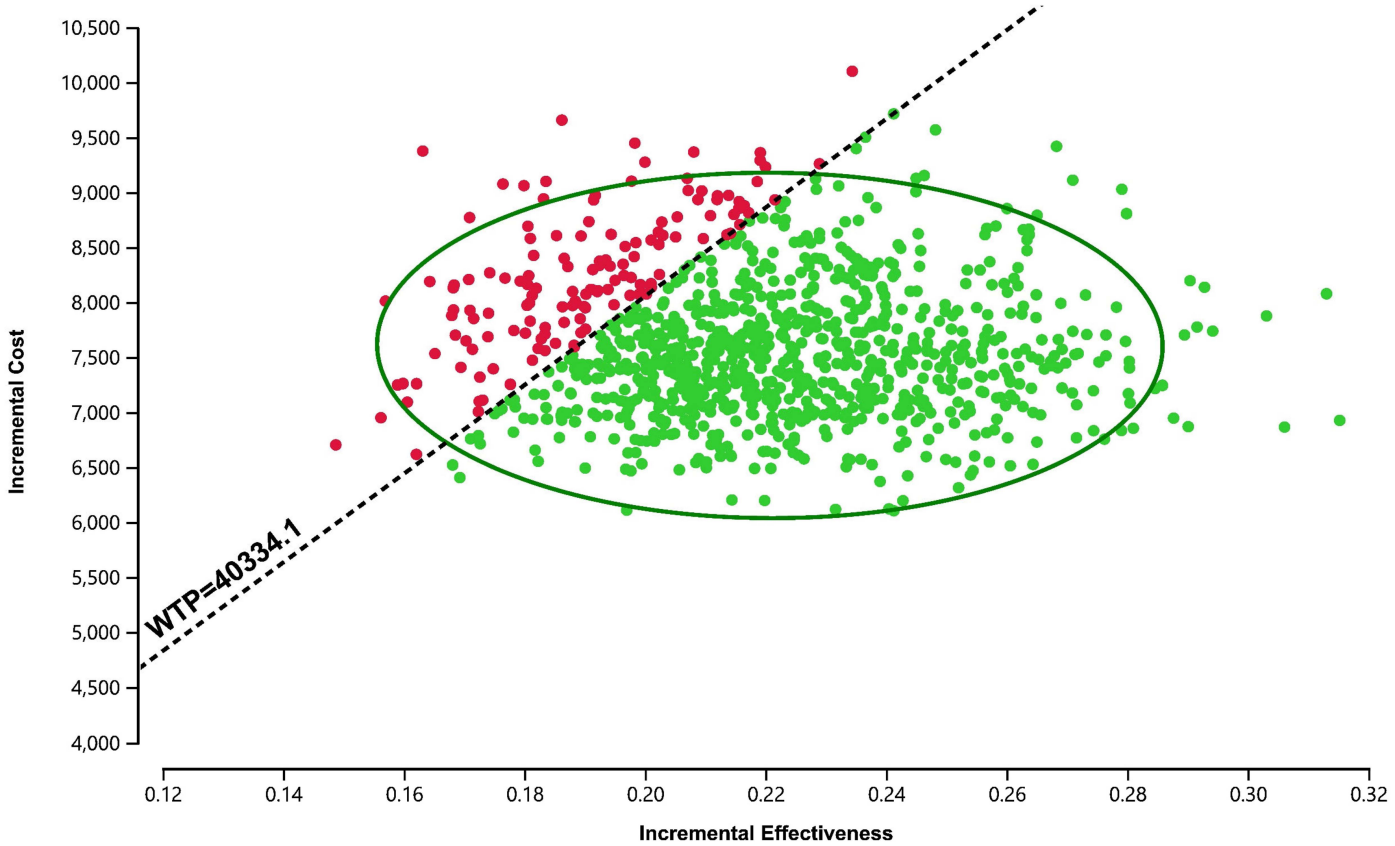
Scatter plot of the probabilistic sensitivity analysis for anlotinib plus penpulimab vs. sorafenib. The black dashed lines indicate the willingness-to-pay (WTP) threshold.

The PSA results were presented via a cost-effectiveness acceptability curve (Figure 3) and a scatter plot (Figure 4). The scatter plot showed that most ICER scatter points were predominantly in the first quadrant, indicating that most patients would achieve improved effectiveness at a higher cost. Notably, 85.9% of the scatter points were below the WTP threshold line in the probabilistic scatter plot, reinforcing the cost-effectiveness of the anlotinib plus penpulimab regimen.

### 3.3 Scenario analysis

The results of the scenario analysis are presented in Table 4. In scenario 1, when the model time horizon was changed to 1, 2, 5, and 10 years, the ICERs of anlotinib plus penpulimab vs. sorafenib were $94896.67, $51697.67, $36967.35, and $34050.28 per QALY, respectively. As the duration increased, the ICER gradually decreased. In scenario 2, when the PAP was excluded, the ICER increased to $46894.83 per QALY, which was above the WTP threshold, indicating that the regimen ceased to be cost-effective under this scenario.

**Table 4:** Results of scenario analyses.

Time horizon	Treatment	Total cost ($)	QALY	Incremental cost ($)	Incremental QALY	ICER ($/QALY)
Scenario 1						
Model runtime (10 years)	Anlotinib plus penpulimab	18082.48	1.19			
	Sorafenib	25681.69	1.42	7599.21	0.22	34050.28
Model runtime (5 years)	Anlotinib plus penpulimab	16838.49	1.09			
	Sorafenib	24393.38	1.29	7554.89	0.20	36967.35
Model runtime (2 years)	Anlotinib plus penpulimab	13187.24	0.79			
	Sorafenib	20938.43	0.94	7751.19	0.15	51697.67
Model runtime (1 year)	Anlotinib plus penpulimab	9811.04	0.52			
	Sorafenib	17974.10	0.61	8163.06	0.09	94896.67
Scenario 2						
Penpulimab without PAP	Anlotinib plus penpulimab	28548.29	1.19			
	Sorafenib	18082.48	1.42	10465.81	0.22	46894.83

QALY, quality-adjusted life year; ICER, incremental cost-effectiveness ratio.

## 4 Discussion

Hepatocellular carcinoma (HCC), a highly prevalent and aggressive malignancy, constitutes a substantial global health burden, especially in advanced stages where curative interventions remain elusive for most patients (29, 30). In recent years, the treatment landscape has undergone transformative shifts with the introduction of tyrosine kinase inhibitors such as sorafenib and lenvatinib, immune checkpoint inhibitors, and, most recently, multi-pathway combination therapies (6, 31, 32). While ICI-based combinations have improved clinical outcomes, they introduce complex cost–benefit considerations that vary substantially across healthcare systems.

To contextualize our findings, we reviewed 28 pharmacoeconomic studies on combination therapies as first-line treatment for advanced HCC (Supplementary Table S5). After excluding network meta-analyses, systematic reviews, and real-world analyses, we identified 18 relevant studies. Of these 18, 10 evaluated atezolizumab plus bevacizumab for advanced HCC across various healthcare systems, including those of the US, France, Thailand, Singapore, and China (23, 33–40). In China, two analyses showed widely varying incremental costs, incremental effectiveness, and ICERs: $156209 and $77139.49, 0.53 and 0.53, and $322500 and $145546.21 per QALY, respectively (34, 37). Two studies found sintilimab plus bevacizumab or its biosimilar to be cost-effective with low ICERs; the incremental costs, incremental effectiveness, and ICERs were $10472 and $12065, 0.500 and 0.493, and $20968 per QALY and $24462 per QALY, respectively (41, 42). Four Chinese studies and one US study supported the cost-effectiveness of camrelizumab plus rivoceranib, with incremental costs, incremental effectiveness, and ICERs in China ranging from $7329.75 to $19569.54, 0.240 to 0.800, and $9147.01 to $33619.98 per QALY, respectively (43–47). Only two studies, both from the US perspective, reported favorable cost-effectiveness for tremelimumab plus durvalumab (48, 49). However, no studies have investigated the economic implications of anlotinib plus penpulimab as a first-line treatment for unresectable HCC.

Recently, the combination of penpulimab and anlotinib demonstrated remarkable efficacy and safety in the APOLLO trial involving patients with unresectable HCC (17). Penpulimab is a novel humanized immunoglobulin G1 (IgG1) anti-PD-1 antibody characterized by high binding affinity and enhanced structural stability (15), and it has demonstrated efficacy both as monotherapy and in combination regimens across various cancer types (50, 51). In contrast to bevacizumab, anlotinib is a small-molecule multi-target tyrosine kinase inhibitor (TKI) with a broader spectrum of kinase inhibition (16). Anlotinib’s suppression of pro-angiogenic signaling (e.g., VEGFR2/PI3K/AKT) and enhancement of TFRC-dependent CD8 + T-cell recruitment may contribute to its sustained efficacy and durability (52, 53). Synergizing with immunotherapy, anlotinib promotes tumor vascular normalization, reprograms the immunosuppressive microenvironment, and enhances intratumoral delivery of immune cells, thereby underpinning its sustained efficacy advantage over sorafenib (54).

Our economic model demonstrated that anlotinib plus penpulimab is potentially cost-effective compared to sorafenib from the perspective of the Chinese healthcare system, with an ICER of $34050.28 per QALY-15.6% below the predefined WTP threshold. Comparative analysis positions this regimen as economically superior to atezolizumab-bevacizumab combinations while matching the cost-effectiveness of sintilimab-bevacizumab biosimilar and camrelizumab plus rivoceranib strategies from the Chinese healthcare perspective. A key influencing factor is likely the lower cost of anlotinib plus penpulimab compared to atezolizumab plus bevacizumab. Previous cost-effectiveness studies of atezolizumab and bevacizumab reported per-cycle costs exceeding $4638.01 and $1908.90, respectively (34, 37). In contrast, the per-cycle costs of anlotinib and penpulimab are $564.20 and $603.79, respectively. This substantial difference in drug pricing may lead to significantly higher healthcare expenditures associated with the atezolizumab–bevacizumab regimen.

The model showed robust stability across sensitivity analyses, identifying treatment duration and PFS utility values as critical drivers of cost-effectiveness. Anlotinib treatment duration exceeding 11.6 cycles or PFS utility values below 0.637 would compromise cost-effectiveness. In the APOLLO trial, the median treatment duration for anlotinib was 10 cycles, compared to sorafenib’s 4 cycles; however, detailed proportions of patients with durations ranging between 4 and 16 cycles were not provided. Since the original APOLLO trial did not include quality-of-life (QoL) measurements, utilities were derived from published studies that closely match our trial population and methodology. This approach could introduce bias into the ICER calculations. However, sensitivity analyses confirmed that this approach did not introduce significant bias, supporting the appropriateness and conservativeness of using these values. Moreover, variations in the WTP threshold can affect ICER interpretations (55, 56). Nevertheless, the model remained robust, with maximum ICERs remaining below the WTP threshold within the predetermined range of other influential factors. Probabilistic sensitivity analysis confirmed this resilience, showing an 85.9% probability of cost-effectiveness at the WTP threshold. These findings suggest the regimen’s economic sustainability despite methodological limitations.

In addition, scenario analyses were conducted to assess two real-world clinical situations, strengthening the relevance and generalizability of the study findings. In scenario 1, more than 70% of the total costs associated with anlotinib plus penpulimab were incurred within the first 2 years. As the treatment time horizon extended, a consistent decrease in ICER values was observed, indicating enhanced cost-effectiveness with prolonged treatment duration. This highlights the importance of treatment adherence, as continued therapy leads to more favorable economic outcomes over time. In scenario 2, the analysis revealed that excluding the PAP, particularly with rising penpulimab costs, resulted in an ICER that exceeded the WTP threshold. This outcome is consistent with sensitivity analyses identifying penpulimab cost as a significantly influential factor, further underscoring the PAP as a key element in the economic profile of the anlotinib plus penpulimab regimen.

This analysis has several limitations. First, as with many cost-effectiveness studies, it relies on clinical trial data derived from populations under strict eligibility criteria, which may limit the generalizability of findings to broader, real-world HCC populations and potentially overestimate efficacy. Second, due to the absence of QoL data in the APOLLO trial, utility values were sourced from external studies, which may not fully reflect the health-state preferences specific to the anlotinib plus penpulimab regimen, potentially affecting the reliability of QALY estimates. Third, although sensitivity analyses suggest that excluding rare adverse events may not significantly alter the outcomes, it might still lead to a slight underestimation of total management costs or utility reductions, warranting caution in resource allocation decisions. Fourth, regional price variations across different provinces in China and the lack of uniform pricing indicate that the use of cost data from Guangdong may introduce bias. Notably, this analysis does not incorporate broader socioeconomic factors, particularly indirect costs such as productivity losses (e.g., income reduction, job loss, and caregiver burden) and significant out-of-pocket expenses. This omission overlooks the substantial financial toxicity experienced by HCC patients in China, a key barrier to accessing even clinically and economically viable therapies such as anlotinib plus penpulimab. Evidence from HCC-specific financial burden studies underscores that these indirect costs are a major driver of household economic hardship and treatment abandonment (57). Future studies should prioritize real-world validation of quality-of-life metrics, long-term survival outcomes, and socioeconomic barriers to optimize implementation strategies and improve model accuracy. There is a clear need for real-world data on treatment adherence, long-term toxicities, and patient-reported outcomes to support equitable policy-making.

To the best of our knowledge, this study is the first to evaluate the cost-effectiveness of anlotinib plus penpulimab as a first-line therapy for unresectable HCC from the perspective of the Chinese healthcare system. Economic simulations show that it is a potentially cost-effective treatment strategy for advanced HCC, supported by factors such as its good tolerability, longer treatment duration, superior clinical outcomes, including overall survival, and relatively low costs compared to other ICIs and anti-angiogenic inhibitors. These findings broaden the first-line options for advanced HCC and provide data to support guidance in the selection of cost-effective ICI regimens.

## 5 Conclusion

Our analysis demonstrates that anlotinib plus penpulimab is likely to provide economic benefits compared with sorafenib for advanced HCC from a Chinese payer’s perspective. It offers evidence to guide the selection of immunotherapeutic regimens and supports decision-makers in optimizing treatment strategies for advanced HCC. These findings highlight the potential of innovative combinations to balance clinical efficacy and economic viability, addressing key socioeconomic challenges in healthcare accessibility. They also underscore the necessity for sustained collaboration among policymakers, healthcare providers, and pharmaceutical stakeholders.

## Data Availability

The raw data supporting the conclusions of this article will be made available by the authors, without undue reservation.
