# Supplementary material for: Economic evaluation of anlotinib plus penpulimab vs. sorafenib as first-line therapy for unresectable hepatocellular carcinoma in China
Source: Front Public Health. 2025 Dec 1;13:1634266. doi: 10.3389/fpubh.2025.1634266 (PMC12702908; doi:10.3389/fpubh.2025.1634266)
Supplement: Supplementary file 1 [file Data_Sheet_1.PDF]

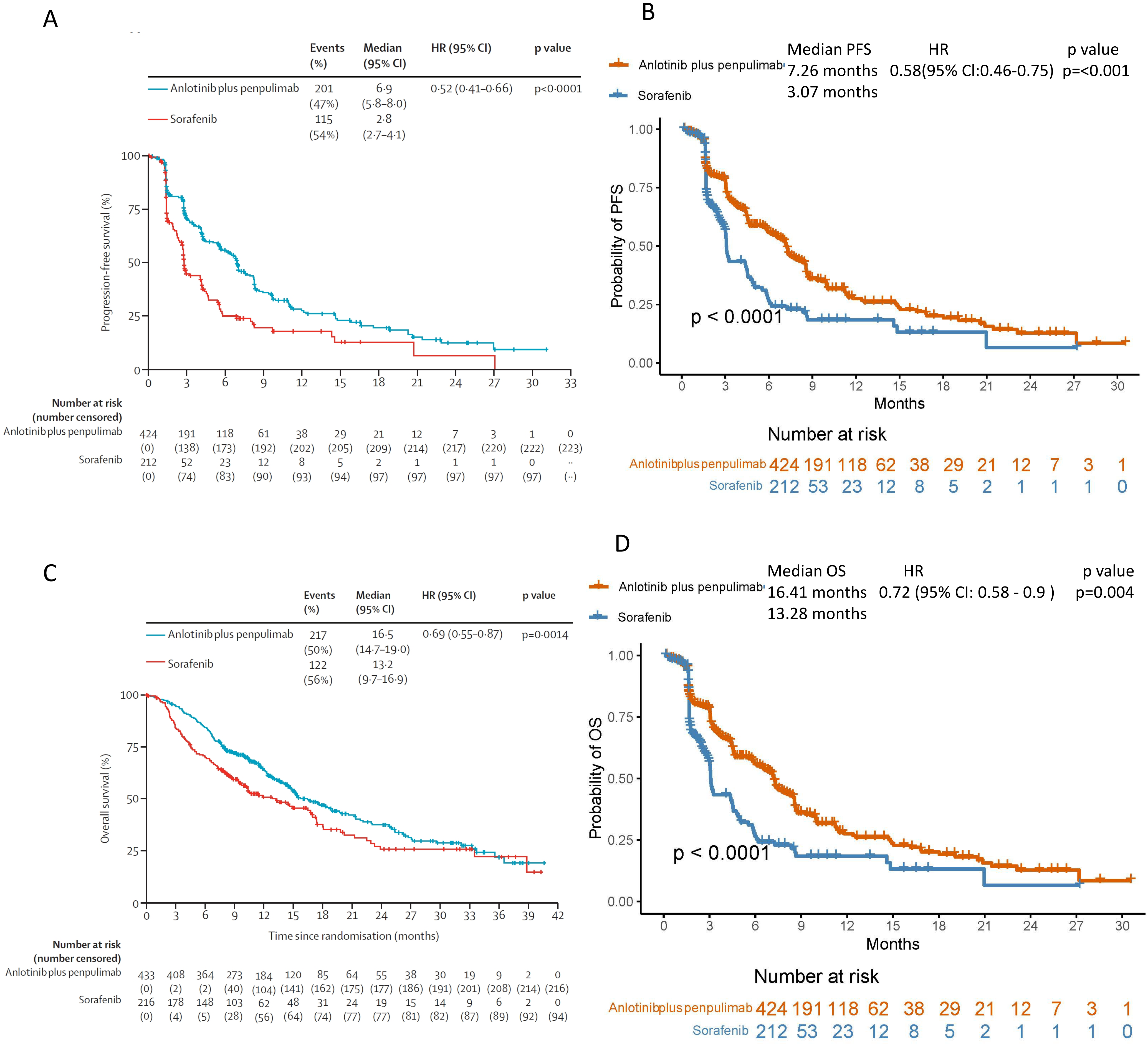

Supplementary Fig S1 The survival curve simulation results.
(A)Kaplan-Meier curve of the PFS from the APOLLO trial.
(B)Simulate PFS curve for Anlotinib plus penpulimab group and Sorafenib group.
(C)Kaplan–Meier curve of OS from the APOLLO trial.
(D)Simulate OS curve for Anlotinib plus penpulimab group and Sorafenib group.
PFS, progression-free survival; OS, over all survival
