# Supplementary material for: Economic evaluation of anlotinib plus penpulimab vs. sorafenib as first-line therapy for unresectable hepatocellular carcinoma in China
Source: Front Public Health. 2025 Dec 1;13:1634266. doi: 10.3389/fpubh.2025.1634266 (PMC12702908; doi:10.3389/fpubh.2025.1634266)
Supplement: Supplementary file 2 [file Data_Sheet_2.PDF]

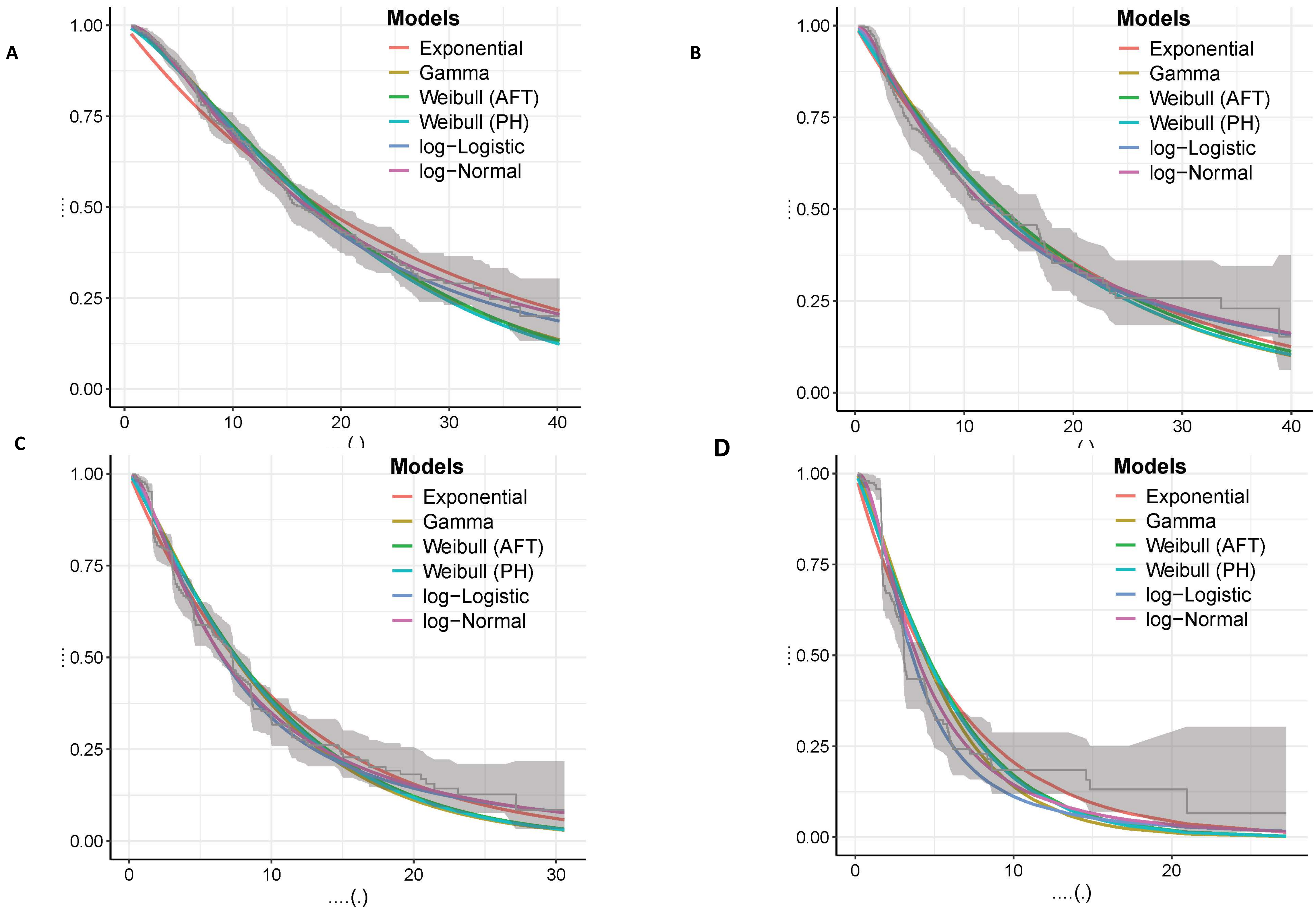

Supplementary Fig S2 Summary of Estimated curves fitted to PFS and OS.
(A)Fitting and extrapolation of the PFS curves in the Anlotinib plus penpulimab group;
(B)Fitting and extrapolation of the PFS curves in the Sorafenib group;
(C)Fitting and extrapolation of the OS curves in the Anlotinib plus penpulimab group;
(D)Fitting and extrapolation of the OS curves in the Sorafenib group;
PFS, progression-free survival, OS, overall survival
