# Supplementary material for: Economic evaluation of anlotinib plus penpulimab vs. sorafenib as first-line therapy for unresectable hepatocellular carcinoma in China
Source: Front Public Health. 2025 Dec 1;13:1634266. doi: 10.3389/fpubh.2025.1634266 (PMC12702908; doi:10.3389/fpubh.2025.1634266)
Supplement: Supplementary file 3 [file Table_1.DOCX]

Supplementary Tables S1 Baseline characteristics of the patients in the APPLO trial

|  | **Anlotinib plus penpulimab group (n=433)** | **Sorafenib group (n=216)** |
| --- | --- | --- |
| Age, years | 57 (50–65) | 56 (50–65) |
| <65 | 315 (73%) | 161 (75%) |
| ≥65 | 118 (27%) | 55 (25%) |
| Sex | | |
| Male | 371 (86%) | 180 (83%) |
| Female | 62 (14%) | 36 (17%) |
| Ethnicity | | |
| Chinese | 433 (100%) | 216 (100%) |
| ECOG performance status score | | |
| 0 | 247 (57%) | 122 (56%) |
| 1 | 186 (43%) | 94 (44%) |
| Baseline α-fetoprotein concentration, ng/mL | | |
| <400 | 220 (51%) | 110 (51%) |
| ≥400 | 213 (49%) | 106 (49%) |
| Barcelona Clinic Liver Cancer stage* | | |
| B | 79/432(18%) | 42 (19%) |
| C | 353/432 (82%) | 174 (81%) |
| Child-Pugh score* | | |
| A | 399/432 (92%) | 201 (93%) |
| B | 33/432 (8%) | 15 (7%) |
| Disease aetiology | | |
| Hepatitis B virus positive | 365 (84%) | 181 (84%) |
| Hepatitis C virus positive | 16 (4%) | 7 (3%) |
| Macrovascular invasion or extrahepatic metastasis | 348 (80%) | 173 (80%) |
| Macrovascular invasion | 179 (41%) | 87 (40%) |
| Extrahepatic metastasis | 267 (62%) | 137 (63%) |
| Both | 98 (23%) | 51 (24%) |
| Previous local treatment for hepatocellular carcinoma | 247 (57%) | 113 (52%) |
| Interventional therapy | 150 (35%) | 70 (32%) |
| Surgery | 118 (27%) | 55 (25%) |
| Ablation | 52 (12%) | 25 (12%) |
| Number of organ sites with target lesions | | |
| One | 301 (70%) | 145 (67%) |
| Two | 107 (25%) | 62 (29%) |
| Three or more | 24 (6%) | 9 (4%) |
| Sum of target lesions size, mm | 89 (49–141) | 90 (53–144) |
| Albumin–bilirubin grade | | |
| 1 | 267 (62%) | 142 (66%) |
| 2 | 166 (38%) | 74 (34%) |

Data are median (IQR) or n (%). ECOG=Eastern Cooperative Oncology Group. *432 patients were included due to one patient with melanoma being incorrectly enrolled.
