# Supplementary material for: Economic evaluation of anlotinib plus penpulimab vs. sorafenib as first-line therapy for unresectable hepatocellular carcinoma in China
Source: Front Public Health. 2025 Dec 1;13:1634266. doi: 10.3389/fpubh.2025.1634266 (PMC12702908; doi:10.3389/fpubh.2025.1634266)
Supplement: Supplementary file 4 [file Table_2.DOCX]

Supplementary Tables S2 Summary of estimated model parameters fitted to PFS and OS in the APOLLO trials

|  | Exponential | gamma | Weibull (AFT) | Weibull (PH) | Log-logistic | Lognormal |
| --- | --- | --- | --- | --- | --- | --- |
| **Anlotinib plus penpulimab OS curve** | | | | | | |
| AIC | 1829.02 | 1803.607 | 1808.971 | 1808.971 | 1796.902 | 1261.074 |
| BIC | 1833.091 | 1811.749 | 1817.112 | 1817.112 | 1805.043 | 1269.173 |
| **Sorafenib OS curve** | | | | | | |
| AIC | 953.0558 | 953.128 | 954.5055 | 954.5055 | 942.5052 | 780.9005 |
| BIC | 956.4311 | 959.8785 | 961.2561 | 961.2561 | 949.2558 | 787.9986 |
| **Anlotinib plus penpulimab PFS curve** | | | | | | |
| AIC | 1298.423 | 1288.64 | 1294.046 | 1294.046 | 1270.213 | 657.4989 |
| BIC | 1302.473 | 1296.739 | 1302.146 | 1302.146 | 1278.312 | 664.5971 |
| **Sorafenib PFS curve** | | | | | | |
| AIC | 577.9444 | 566.2558 | 574.1232 | 574.1232 | 536.5154 | 646.6346 |
| BIC | 581.3009 | 572.969 | 580.8363 | 580.8363 | 543.2286 | 653.7328 |

OS, overall survival; PFS, progression-free survival; AIC, Akaike’s information criterion; BIC, Bayesian information criterion
