# Supplementary material for: Economic evaluation of anlotinib plus penpulimab vs. sorafenib as first-line therapy for unresectable hepatocellular carcinoma in China
Source: Front Public Health. 2025 Dec 1;13:1634266. doi: 10.3389/fpubh.2025.1634266 (PMC12702908; doi:10.3389/fpubh.2025.1634266)
Supplement: Supplementary file 5 [file Table_3.DOCX]

Supplementary Table S3 A summary of duration and dose of each therapy.

| Variables | Base value | Range | | Distribution | Source |
| --- | --- | --- | --- | --- | --- |
|  |  | Minimum | Maximum |  |  |
| **Duration of the therapy (cycle)** | | | | | |
| Duratio of Penpulimab | 10.00 | 8.00 | 12.00 | Gamma | [1] |
| Duration of Anlotinib | 10.00 | 8.00 | 12.00 | Gamma | [1] |
| Duration of Sorafenib | 4.00 | 3.20 | 4.80 | Gamma | [1] |
| **Dose of the therapy (mg/cycle)** | | | | | |
| Dose of Penpulimab | 200.00 | 160.00 | 240.00 | Gamma | [1] |
| Dose of Anlotinib | 140.00 | 112.00 | 168.00 | Gamma | [1] |
| Dose of Sorafenib | 16800.00 | 13440.00 | 20160.00 | Gamma | [1] |

1. Zhou J, Bai L, Luo J, Bai Y, Pan Y, Yang X, Gao Y, Shi R, Zhang W, Zheng J *et al*: **Anlotinib plus penpulimab versus sorafenib in the first-line treatment of unresectable hepatocellular carcinoma (APOLLO): a randomised, controlled, phase 3 trial**. *Lancet Oncol* 2025.
