# Supplementary material for: Economic evaluation of anlotinib plus penpulimab vs. sorafenib as first-line therapy for unresectable hepatocellular carcinoma in China
Source: Front Public Health. 2025 Dec 1;13:1634266. doi: 10.3389/fpubh.2025.1634266 (PMC12702908; doi:10.3389/fpubh.2025.1634266)
Supplement: Supplementary file 6 [file Table_4.DOCX]

Supplementary Table S4 Treatment-related adverse events of Grade≥3 in APOLLO in the safety population

| **Anlotinib plus penpulimab (n=432)** | | | | **Sorafenib (n=211)** | | | | |
| --- | --- | --- | --- | --- | --- | --- | --- | --- |
|  | Grade 3 | Grade 4 | Grade 5 | | Grade 3 | Grade 4 | Grade 5 | |
| Any treatment-related adverse event | 188 (44%) | 26 (6%) | 1 (<1%) | | 89 (42%) | 10 (5%) | | 2 (1%) |
| Hypertension | 75 (17%) | 0 | 0 | | 22 (10%) | 0 | | 0 |
| Platelet count decreased | 33 (8%) | 6 (1%) | 0 | | 13 (6%) | 0 | | 0 |
| Aspartate aminotransferase concentrations increased | 16 (4%) | 2 (<1%) | 0 | | 12 (6%) | 1 (<1%) | | 0 |
| Blood bilirubin concentrations increased | 16 (4%) | 7 (2%) | 0 | | 5 (2%) | 0 | | 0 |
| White blood cell count decreased | 23 (5%) | 1 (<1%) | 0 | | 7 (3%) | 0 | | 0 |
| Alanine aminotransferase concentrations increased | 10 (2%) | 0 | 0 | | 7 (3%) | 0 | | 0 |
| Hypothyroidism | 0 | 0 | 0 | | 0 | 0 | | 0 |
| Neutrophil count decreased | 24 (6%) | 1 (<1%) | 0 | | 5 (2%) | 2 (1%) | | 0 |
| Palmar-plantar erythrodysesthesia syndrome | 8 (2%) | 0 | 0 | | 17 (8%) | 0 | | 0 |
| Hypoalbuminaemia | 3 (1%) | 0 | 0 | | 0 | 0 | | 0 |
| Weight loss | 2 (<1%) | 0 | 0 | | 3 (1%) | 0 | | 0 |
| Proteinuria | 3 (1%) | 0 | 0 | | 0 | 0 | | 0 |
| Diarrhea | 14 (3%) | 0 | 0 | | 2 (1%) | 0 | | 0 |
| Thyroid-stimulating hormone concentrations increased | 0 | 0 | 0 | | 0 | 0 | | 0 |
| Anaemia | 9 (2%) | 0 | 0 | | 3 (1%) | 1 (<1%) | | 0 |
| Lymphocyte count decreased | 17 (4%) | 0 | 0 | | 4 (2%) | 0 | | 0 |
| Gamma-glutamyltransferase concentrations increased | 7 (2%) | 0 | 0 | | 5 (2%) | 0 | | 0 |
| Fatigue | 1 (<1%) | 0 | 0 | | 2 (1%) | 0 | | 0 |
| Occult blood positive | 0 | 0 | 0 | | 0 | 0 | | 0 |
| Anorexia | 2 (<1%) | 0 | 0 | | 1 (<1%) | 0 | | 0 |
| Alkaline phosphatase concentrations increased | 1 (<1%) | 0 | 0 | | 2 (1%) | 0 | | 0 |
| Rash | 2 (<1%) | 0 | 0 | | 2 (1%) | 0 | | 0 |
| Hypokalaemia | 10 (2%) | 1 (<1%) | 0 | | 3 (1%) | 3 (1%) | | 0 |
