# Supplementary material for: Economic evaluation of anlotinib plus penpulimab vs. sorafenib as first-line therapy for unresectable hepatocellular carcinoma in China
Source: Front Public Health. 2025 Dec 1;13:1634266. doi: 10.3389/fpubh.2025.1634266 (PMC12702908; doi:10.3389/fpubh.2025.1634266)
Supplement: Supplementary file 7 [file Table_5.DOCX]

Supplementary Table S5

The detailed direct cost results of the cost-effectiveness analysis

| Direct cost ($) | Anlotinib plus Penpulimab | Sorafenib |
| --- | --- | --- |
| Penpulimab | 3348.35 |  |
| Anlotinib | 4299.97 |  |
| Subsequent systemic therapy in Anlotinib | 4289.51 |  |
| Sorafenib |  | 1748.59 |
| Subsequent systemic therapy in sorafenib |  | 6852.71 |
| Administration cost | 907.29 | 419.63 |
| Cost of laboratory tests and radiological examinations | 4785.88 | 2213.48 |
| Routine follow-up cost | 1157.84 | 830.78 |
| Best supportive care | 4310.28 | 4232.86 |
| End-of-life care | 1351.37 | 1366.34 |
| Costs of serious adverse events | 1231.19 | 418.05 |
| Total cost | 25681.69 | 18082.48 |
