# Supplementary material for: Economic evaluation of anlotinib plus penpulimab vs. sorafenib as first-line therapy for unresectable hepatocellular carcinoma in China
Source: Front Public Health. 2025 Dec 1;13:1634266. doi: 10.3389/fpubh.2025.1634266 (PMC12702908; doi:10.3389/fpubh.2025.1634266)
Supplement: Supplementary file 8 [file Table_6.DOCX]

Supplementary Table S6 The literature of pharmacoeconomic studies on combination therapies as a first-line treatment for advanced HCC

| No | Intervention Group | Control Group | Incremental cost ($) | Incremental QALY | ICER ($/QALY) | WTP ($per QALY) | Healthcare system | Result | Study type | **Source** |
| --- | --- | --- | --- | --- | --- | --- | --- | --- | --- | --- |
| 1 | Sintilimab - IBI305 | lenvatinib | 12065 | 0.493 | 24462 | 37547 | China | Cost-effective | CEA | (1) |
| 2 | Atezolizumab - Bevacizumab | Sorafenib | 89807 | 0.530 | 169223 | 150000 | US | Not cost-effective | CEA | (2) |
| 3 | Atezolizumab - Bevacizumab | Sorafenib | 156209 | 0.484 | 322500 | 100000 | China | Not cost-effective | CEA | (3) |
| 4 | Atezolizumab - Bevacizumab | Sorafenib | 99174 | 0.600 | 163651 | 133775 | France | Not cost-effective | CEA | (4) |
| 5 | Atezolizumab - Bevacizumab | Sorafenib | 79074 | 0.439 | 179729 | 150000 | China Hongkong | Not cost-effective | CEA | (5) |
| 6 | Atezolizumab - Bevacizumab | Nivolumab | 78280.00 | 0.680 | 113892 | 150000 | US | Cost-effective | CEA | (6) |
| 7 | Atezolizumab - Bevacizumab | Sorafenib | 77139.49 | 0.530 | 145546.21 | 28527 | China | Not cost-effective | CEA | (7) |
| 7 | Atezolizumab - Bevacizumab | Sorafenib | 89056.01 | 0.530 | 168030.21 | 150000 | US | Not cost-effective | CEA |  |
| 8 | Sintilimab - Bevacizumab | Sorafenib | 10472 | 0.500 | 20968 | 33592 | China | Cost-effective | CEA | (8) |
| 9 | Atezolizumab - Bevacizumab | Best supportive care | 45357 | 0.435 | 54589 | 4678 | Thailand | Not cost-effective | CEA | (9) |
| 10 | Camrelizumab-Rivoceranib | Sorafenib | 13684.84 | 0.410 | 33619.98 | 35864.61 | China | Cost-effective | CEA | (10) |
| 11 | Camrelizumab-rivoceranib | sorafenib | 7345.05 | 0.80 | 9147.01 | 39 855.785 | China. | cost-effective | CEA | (11) |
| 12 | atezolizumab-bevacizumab | sorafenib | 83887.49 | 0.82 | 83887.49 | 85626.03 | Singapore | cost-effective | CEA | (12) |
| 13 | durvalumab-tremelimumab | sorafenib | -30179.00 | 0.29 | -106307.00 |  | USA | cost-effective | CEA | (13) |
| 14 | atezolizumab-bevacizumab | sorafenib | 127607.00 | 1.70 | 75192.00 | 92480.00 | ChinaTaiwan | cost-effective | CEA | (14) |
| 15 | camrelizumab and rivoceranib | sorafenib | 86994.21 | 0.71 | 122388.62 | 150000.00 | US | cost-effective | CEA | (15) |
| 15 | camrelizumab and rivoceranib | sorafenib | 19569.54 | 0.64 | 30410.56 | 35898.87 | China | cost-effective | CEA |  |
| 16 | Camrelizumab-Rivoceranib | Rivoceranib | 8340.00 | 0.24 | 34 897 | 36 780 | China | cost-effective | CEA | (16) |
| 17 | Tremelimumab-Durvalumab | sorafenib | 19256.49 | 0.20 | 97995.51 | 150000.00 | USA | cost-effective | CEA | (17) |
| 18 | camrelizumab-rivoceranib |  | 7329.75. | 0.80 | 9150.75 | low-income ($16426.80) medium-income ($34319.01)  high-income regions ($81036.63). | China | cost-effective | CEA | (18) |
| 19 | Sintilimab - Bevacizumab | Sorafenib | 26580.35 | 0.510 | 51877.36 | 33521 | China | Not cost-effective | Network meta-analysis | (19) |
|  | Atezolizumab - Bevacizumab | Sorafenib | 112666.86 | 0.860 | 130508.44 |  | China | Not cost-effective |  |  |
| 20 | Atezolizumab - Bevacizumab | Donafenib | 39139.87 | 0.460 | 85607.88 | 11101.70 | China | Not cost-effective | Network meta-analysis | (20) |
|  | Sintilimab - Bevacizumab | Donafenib | 15093.04 | 1.250 | 12109.27 |  | China | Not cost-effective |  |  |
|  | Atezolizumab - Bevacizumab | Nivolumab | 179938.7 | 3.750 | 47896.93 | 69375 | US | Cost-effective |  |  |
| 21 | Sintilimab - IBI305 | Sorafenib | 24542.33 | 0.620 | 39766.86 | 33500 | China | Not cost-effective | Network meta-analysis | (21) |
|  | Atezolizumab - Bevacizumab | Sorafenib | 61397.35 | 0.600 | 103037.66 |  | China | Not cost-effective |  |  |
| 22 | Cabozantinib - Atezolizumab | Sorafenib | 27650 | 0.121 | 228512 | 37653 | China | Not cost-effective | Network meta-analysis | (22) |
|  | Pembrolizumab - Lenvatinib | Sorafenib | 15985 | 0.305 | 52410 |  | China | Not cost-effective |  |  |
|  | Camrelizumab - Rivoceranib | Sorafenib | 11561 | 0.506 | 22848 |  | China | Cost-effective |  |  |
|  | Atezolizumab - Bevacizumab | Sorafenib | 44711 | 0.581 | 76955 |  | China | Not cost-effective |  |  |
|  | Sintilimab - IBI305 | Sorafenib | 27513 | 0.787 | 34959 |  | China | Cost-effective |  |  |
| 23 | Sintilimab-Bevacizumab | Sorafenib | 23296.6 | 0.310 | 75150.32 | 36600 | China | Not cost-effective | Network meta-analysis | (23) |
|  | Atezolizumab-Bevacizumab | Sorafenib | 125726.93 | 0.870 | 144513.71 |  | China | Not cost-effective |  |  |
| 24 | atezolizumab-bevacizumab | sorafenib | NA | NA | 196 704 | 150 000 | US | Not cost-effective | Network meta-analysis | (24) |
|  | tremelimumab-durvalumab | sorafenib | NA | NA | 800 755 |  | US | Not cost-effective |  |  |
| 25 | sintilimab-IBI305 | sorafenib | 74083.00 | 0.85 | 115760 | 37654.50 | China | Not cost-effective | Network meta-analysis | (25) |
|  | atezolizumab-bevacizumab | sorafenib | 104188 and | 0.84 | 160049 | 37654.50 | China | Not cost-effective |  |  |
| 26 | tremelimumab-durvalumab (STRIDE) | NA | 84589.01 | 0.73 | NA | 73500.00 | China |  | Network meta-analysis | (26) |
|  | atezolizumab-bevacizumab (A + B) | NA | 70985.06 | 0.90 | NA |  | China |  |  |  |
|  | sintilimab-bevacizumab biosimilar (IBI305) (S + B) | NA | 26961.60 | 1.12 | 24072.86 |  | China | Cost-effective |  |  |
|  | camrelizumab-rivoceranib (C + R) | NA | 12109.27 | 0.91 | 13306.89 |  | China | Not cost-effective |  |  |
|  | pembrolizumab-lenvatinib (P + L). | NA | 26961.60 | 1.12 | NA |  | China |  |  |  |
| 27 | durvalumab - tremelimumab | NA | NA | NA | NA | NA | NA | NA | Systematic Review | (27) |
| 28 | sintilimab-bevacizumab | NA | 35018 | 2.92 | NA | 37547 | China | cost-effectiveness | Real-world analysis | (28) |

CEA: Cost-Effectiveness Analysis; QALY: quality-adjusted life-year; ICER: incremental cost effectiveness ratio; IBI305: bevacizumab biosimilar; USD: United States Dollar;

**Reference:**

1. Zhou T Wang X Cao Y Yang L Wang Z Ma A et al. Cost-effectiveness analysis of sintilimab plus bevacizumab biosimilar compared with lenvatinib as the first-line treatment of unresectable or metastatic hepatocellular carcinoma. BMC Health Serv Res. 2022;22(1):1367.

2. Su D Wu B Shi L. Cost-effectiveness of Atezolizumab Plus Bevacizumab vs Sorafenib as First-Line Treatment of Unresectable Hepatocellular Carcinoma. JAMA Netw Open. 2021;4(2):e210037.

3. Zhang X Wang J Shi J Jia X Dang S Wang W. Cost-effectiveness of Atezolizumab Plus Bevacizumab vs Sorafenib for Patients With Unresectable or Metastatic Hepatocellular Carcinoma. JAMA Netw Open. 2021;4(4):e214846.

4. Gaugain L Cawston H Dubois de Gennes C Sanchez Alvares J Nahon P Mazaleyrat B et al. Cost-utility analysis of atezolizumab with bevacizumab in untreated unresectable or advanced hepatocellular carcinoma in France. PLoS One. 2023;18(1):e0280442.

5. Chiang CL Chan SK Lee SF Choi HC. First-Line Atezolizumab Plus Bevacizumab versus Sorafenib in Hepatocellular Carcinoma: A Cost-Effectiveness Analysis. Cancers (Basel). 2021;13(5).

6. Li Y Liang X Li H Chen X. Atezolizumab plus bevacizumab versus nivolumab as first-line treatment for advanced or unresectable hepatocellular carcinoma: A cost-effectiveness analysis. Cancer. 2022;128(22):3995-4003.

7. Wen F Zheng H Zhang P Liao W Zhou K Li Q. Atezolizumab and bevacizumab combination compared with sorafenib as the first-line systemic treatment for patients with unresectable hepatocellular carcinoma: A cost-effectiveness analysis in China and the United states. Liver Int. 2021;41(5):1097-104.

8. Zhou T Cao Y Wang X Yang L Wang Z Ma A et al. Economic Evaluation of Sintilimab Plus Bevacizumab Versus Sorafenib as a First-line Treatment for Unresectable Hepatocellular Carcinoma. Adv Ther. 2022;39(5):2165-77.

9. Sriphoosanaphan S Pantumongkol W Kulpeng W Charonpongsuntorn C Tanwandee T Sukeepaisarnjaroen W et al. Cost-utility analysis of atezolizumab combined with bevacizumab for unresectable hepatocellular carcinoma in Thailand. PLoS One. 2024;19(3):e0300327.

10. Lang W Deng L Huang B Zhong D Zhang G Lu M et al. Cost-Effectiveness Analysis of Camrelizumab Plus Rivoceranib Versus Sorafenib as a First-Line Therapy for Unresectable Hepatocellular Carcinoma in the Chinese Health Care System. Clin Drug Investig. 2024;44(3):149-62.

11. Cai H Lin J Zhu H Zheng Z. Camrelizumab plus rivoceranib compared sorafenib as first-line therapeutic options for advanced hepatocellular carcinoma in China: a cost-effectiveness analysis. BMJ Open. 2024;14(12):e079603.

12. Chee CE Khara JS Cheong J Fong J Sivanesan S Choy JY et al. Cost-effectiveness analysis of atezolizumab plus bevacizumab compared with sorafenib as first-line treatment in advanced hepatocellular carcinoma in Singapore. Expert Rev Pharmacoecon Outcomes Res. 2024;24(5):631-41.

13. Liao W Xu H Hutton D Wu Q Yang Y Feng M et al. Cost-effectiveness analysis of durvalumab plus tremelimumab as first-line therapy in patients with unresectable hepatocellular carcinoma. Ther Adv Med Oncol. 2024;16:17588359241274625.

14. Tseng CY Tsai YW Shiu MN. Cost-effectiveness analysis of atezolizumab plus bevacizumab versus sorafenib in first line treatment for Chinese subpopulation with unresectable hepatocellular carcinoma. Front Oncol. 2023;13:1264417.

15. Wei J Xu K Lin Y Liu Q Zhou C Zhang P et al. Economic evaluation of camrelizumab plus rivoceranib versus sorafenib as first-line therapy for unresectable hepatocellular carcinoma in the United States and China. Int J Clin Pharm. 2024;46(5):1189-99.

16. Xiang G Huang Y Zhang N Du X Wu Y Gan L et al. First-Line Camrelizumab Plus Rivoceranib in Advanced Hepatocellular Carcinoma: A China-Based Cost-Effectiveness Analysis. Clin Med Insights Oncol. 2024;18:11795549241299393.

17. Xiong X Guo JJ. Cost Effectiveness of Tremelimumab Plus Durvalumab for Unresectable Hepatocellular Carcinoma in the USA. Pharmacoeconomics. 2025;43(3):271-82.

18. Zhao Z Jiang X Wen S Hao Y. Cost-effectiveness of camrelizumab plus rivoceranib for advanced hepatocellular carcinoma in the context of regional disparities in China. Front Oncol. 2024;14:1491404.

19. Zhao M Pan X Yin Y Hu H Wei J Bai Z et al. Cost-Effectiveness Analysis of Five Systemic Treatments for Unresectable Hepatocellular Carcinoma in China: An Economic Evaluation Based on Network Meta-Analysis. Front Public Health. 2022;10:869960.

20. Sun KX Cao SS Shi FH Guan Y Tang M Zhao MN et al. First-line treatments for advanced hepatocellular carcinoma: a network meta-analysis and cost-effectiveness analysis in China and the United States. Therap Adv Gastroenterol. 2022;15:17562848221140662.

21. Li L Yang S Chen Y Tian L He Y Wu B et al. Immune Checkpoint Inhibitors Plus an Anti-VEGF Antibody as the First-Line Treatment for Unresectable Hepatocellular Carcinoma: A Network Meta-Analysis and Cost-Effectiveness Analysis. Front Pharmacol. 2022;13:891008.

22. Liu K Zhu Y Zhu H. Immunotherapy or targeted therapy as the first-line strategies for unresectable hepatocellular carcinoma: A network meta-analysis and cost-effectiveness analysis. Front Immunol. 2022;13:1103055.

23. Gong H Ong SC Li F Shen Y Weng Z Zhao K et al. Cost-effectiveness of immune checkpoint inhibitors as a first-line therapy for advanced hepatocellular carcinoma: a systematic review. Health Econ Rev. 2024;14(1):48.

24. Lian D Gan Y Xiao D Xuan D Liu S Wei Y. Cost-effectiveness of first-line systemic therapies for unresectable hepatocellular carcinoma. Br J Clin Pharmacol. 2024.

25. Wang L Peng Y Qin S Wan X Zeng X Li S et al. First-line systemic treatment strategies for unresectable hepatocellular carcinoma: A cost-effectiveness analysis. PLoS One. 2023;18(4):e0279786.

26. Wen F Huang P Wu Q Yang Y Zhou K Zhang M et al. Promising first-line immuno-combination therapies for unresectable hepatocellular carcinoma: A cost-effectiveness analysis. Cancer Med. 2024;13(16):e70094.

27. Mohammadnezhad G Esmaily H Talebi M Jafari M. Atezolizumab and Bevacizumab Targeted-Therapy in Advanced Hepatocellular Carcinoma: A Systematic Review of Cost-effectiveness Analyses. J Gastrointest Cancer. 2024;55(2):625-37.

28. Zeng X Jia Y Chen H Luo Q Zhao H Liang G et al. A real-world analysis of survival and cost-effectiveness of sintilimab plus bevacizumab biosimilar regimen in patients with advanced hepatocellular carcinoma. J Cancer Res Clin Oncol. 2023;149(11):9213-9.
